# Are Fathers Being Left Behind? Gender Differences in Parental HPV Vaccination Knowledge and Attitudes Toward Sons’ Vaccination in Greece

**DOI:** 10.3390/vaccines14050455

**Published:** 2026-05-19

**Authors:** Magdalini Christodoulou, Chrisoula Paraforou, Erasmia Rouka, Aikaterini Toska, Dimitrios Papagiannis

**Affiliations:** 1 Public Health & Adults Immunization Laboratory, Department of Nursing, University of Thessaly, 41500 Larissa, Greece; magvet@yahoo.com; 2 Post Graduate Program in Primary Health Care, School of Health Sciences, University of Thessaly, 41500 Larissa, Greece; paraforouchrysa@gmail.com; 3 School of Health Sciences, Department of Nursing, University of Thessaly, 41500 Larissa, Greece; errouka@uth.gr (E.R.); ktoska@uth.gr (A.T.); 4 Department of Neurology, Medical School, University of Cyprus, Nicosia 2029, Cyprus

**Keywords:** HPV, vaccination, gender differences, parental attitudes, knowledge, Greece

## Abstract

Objectives: Despite the critical role fathers play in family health decisions, most research on HPV vaccination focuses predominantly on mothers. This study examines gender differences in HPV knowledge and vaccination attitudes among Greek parents, addressing a significant gap in the literature. Methods: A cross-sectional study using convenience sampling was conducted in waiting rooms of public primary healthcare settings in the Larissa prefecture of central Greece, between September and December 2024. Of 250 distributed questionnaires, 208 were returned (response rate: 83%), of which 192 were eligible for analysis. The analysis compares responses from fathers (*n* = 42) and mothers (*n* = 150) regarding HPV knowledge, intentions to vaccinate their sons, and general vaccine attitudes; no explicit restriction to one respondent per family was applied. Statistical comparisons employed chi-square tests, Fisher’s exact test, and binary logistic regression. Results: Fathers demonstrated significantly lower HPV awareness compared to mothers (42.9% vs. 64.0%, χ^2^ = 10.907, *p* = 0.004). Vaccination intentions for sons were similar between groups (fathers: 85.7%, mothers: 85.3%, *p* = 0.540). No statistically robust association between HPV awareness and vaccination intention was identified in either group, likely reflecting the high overall intention rates and limited outcome variability. Binary logistic regression identified female sex as the only significant independent predictor of HPV awareness (OR = 2.26, 95% CI: 1.12–4.58, *p* = 0.024). Conclusions: While fathers exhibit significantly lower HPV knowledge than mothers, they demonstrate equal willingness to vaccinate their sons. These findings suggest that knowledge gaps do not necessarily translate to vaccine hesitancy, but highlight the need for targeted, father-inclusive health education interventions. Public health programs should actively engage fathers in HPV vaccination discussions to capitalize on their positive vaccination intentions while addressing their information needs.

## 1. Introduction

Human papillomavirus (HPV) is among the most prevalent sexually transmitted infections (STIs) worldwide, affecting both women and men, with an 80% lifetime risk of infection [1,2]. HPV is responsible for nearly all cases of cervical cancer and other cancers, including oropharyngeal, penile, and rectal malignancies, as well as common non-malignant conditions such as genital warts [3,4]. The burden of HPV-related diseases has prompted widespread vaccination programs, and the Advisory Committee on Immunization Practices (ACIP) recommends routine HPV vaccination at ages 11–12 for both girls and boys [5].

The 9-valent HPV vaccine, approved in both Europe and the United States, protects against nine HPV types (6, 11, 16, 18, 31, 33, 45, 52, and 58), collectively accounting for approximately 90% of cervical cancer cases, 82% of high-grade precancerous lesions in the anorectal region, and 90% of genital warts [6]. Despite documented vaccine efficacy and safety, vaccination rates across Europe remain substantially below the World Health Organization’s 90% target [7]. Several European Union countries have incorporated gender-neutral vaccination into their national immunization programs; however, boys typically have lower vaccination rates than girls [8].

In Greece, the National Immunization Committee recommended HPV vaccination for girls starting in 2008, initially targeting ages 12–26 years and subsequently 11–18 years [9]. Since Spring 2022, Greece has expanded its recommendation to include boys, offering free vaccination to both sexes aged 9–18 years [9]. However, vaccination coverage remains suboptimal, with estimates of 55.4% for girls aged 11–18 years and 43.8% for ages 11–14 years [9]. The COVID-19 pandemic further disrupted vaccination programs, resulting in decreased overall HPV vaccination uptake [10].

The effectiveness of vaccination programs depends critically on parental acceptance, as children receive HPV vaccines during early adolescence when parents serve as primary decision-makers [11,12]. Previous research has identified various factors influencing parental vaccination decisions, including knowledge about HPV and the vaccine, personal beliefs, healthcare provider recommendations, and sociodemographic characteristics [13,14,15]. However, the vast majority of studies examining parental HPV vaccination attitudes focus predominantly on mothers, with fathers either explicitly excluded or significantly underrepresented in study samples [16,17,18].

The underrepresentation of fathers in HPV vaccination research represents a critical gap in the HPV vaccination literature. Fathers play an increasingly active role in family health decisions, yet their perspectives, knowledge gaps, and specific barriers to vaccination acceptance remain poorly understood [19,20]. Recent systematic reviews have called for more research examining fathers’ roles in childhood vaccination decisions, noting that paternal attitudes may differ substantially from maternal attitudes and that both parents influence vaccination outcomes [21,22].

Moreover, the limited research that has included fathers suggests potential gender differences in HPV knowledge and attitudes. Studies from the United Kingdom, United States, and Australia have reported that fathers tend to have lower awareness of HPV and the vaccine than mothers, though findings on vaccination intentions have been inconsistent [23,24,25]. However, to our knowledge, no studies have specifically examined gender differences in parental HPV vaccination attitudes in Greece, where gender-neutral vaccination for boys was only recently implemented.

Understanding gender-specific knowledge gaps and attitudes is essential for developing effective, inclusive public health interventions. If fathers have distinct information needs or face unique barriers to vaccine acceptance, health education programs must be tailored accordingly. Furthermore, given that vaccination decisions often involve both parents, ensuring that fathers have adequate knowledge may be crucial for achieving optimal vaccination coverage.

Therefore, the objective of this study is to examine gender differences in HPV knowledge, vaccination attitudes, and intentions among Greek parents of adolescent boys. Specifically, we aim to: (1) compare fathers’ and mothers’ knowledge about HPV infection and vaccination; (2) assess gender differences in intentions to vaccinate sons against HPV; (3) identify specific knowledge gaps and barriers that may be unique to fathers; and (4) provide evidence-based recommendations for gender-inclusive vaccination promotion strategies.

These findings are particularly relevant in the context of expanding gender-neutral HPV vaccination strategies across Europe, where understanding parental decision-making dynamics is essential for improving vaccine uptake and achieving public health targets. In this context, identifying gender-specific barriers to health information access and tailoring communication strategies accordingly represents a critical priority for national immunization programs seeking to achieve equitable vaccination coverage for both sexes.

## 2. Materials and Methods

### 2.1. Study Design and Setting

The present cross-sectional study was conducted at public primary health centers in the Larissa region of Central Greece between September and December 2024. The study period followed Greece’s expansion of free HPV vaccination for boys (implemented in Spring 2022), providing important baseline data on parental attitudes during this transition.

### 2.2. Participants and Sampling

The study employed convenience sampling methodology. Questionnaires were ad-ministered consecutively to all parents present in the waiting rooms of public primary health centers in the Larissa prefecture during scheduled pediatric and general practice clinic sessions throughout the study period. This setting was selected because it represents a primary point of contact between parents and the healthcare system for children’s preventive care, including vaccination. Parents were eligible to participate if they had at least one son aged 9–18 years and could read and understand Greek. No exclusion criterion was applied based on the number of respondents per family, questionnaires were distributed individually to each attending parent, and it is possible that in a small number of cases, both parents of the same child attended on separate occasions and both participated. A total of 250 questionnaires were distributed to eligible parents. Of these, 208 questionnaires were completed and returned, yielding a response rate of 83%. The current analysis focuses specifically on questionnaires completed by mothers (*n* = 150) or fathers (*n* = 42), excluding those completed by other guardians (*n* = 16), resulting in a final analytical sample of 192 participants. The pronounced gender imbalance observed (fathers: 21.9%; mothers: 78.1%) is consistent with the well-documented pattern of predominantly maternal attendance at pediatric primary care visits in Greece.

Sample size considerations: With 42 fathers and 150 mothers, this study has 80% power to detect a medium effect size (Cohen’s h = 0.40) at α = 0.05, corresponding to a difference of approximately 15–20 percentage points in dichotomous outcomes, which is clinically meaningful for public health interventions.

### 2.3. Study Instrument

Data were collected using an anonymous, modified self-administered questionnaire adapted from previously published instruments in HPV vaccination research, specifically those by Papagiannis et al. (2013) and Toska et al. (2024) [26,27]. Additional modifications were introduced to address fathers’ perspectives and potential father-specific barriers, based on relevant international literature [23,28,29], as well as to reflect the current Greek context, including the 2022 expansion to gender-neutral HPV vaccination. The questionnaire comprised four main sections:

Sociodemographic Information: This section collected data on parental characteristics, including (a) age group (18–30 years, 31–45 years, 46+ years), (b) education level (secondary-gymnasium, secondary-lyceum, university, postgraduate), (c) country of origin, (d) relationship to child (mother, father, other) and (e) place of residence (urban, semi-urban, rural).

HPV Knowledge Assessment: This section assessed knowledge of HPV through items covering transmission, associated diseases, and prevalence. Participants responded using a four-point Likert scale (“strongly agree,” “agree,” “disagree,” “strongly disagree”). Items addressed key concepts such as HPV as a sexually transmitted infection, its association with cervical and other cancers, the availability of screening and vaccination, and common misconceptions regarding HPV prevention.

HPV Vaccination Knowledge and Intentions: This section evaluated (a) general awareness of HPV and related diseases, (b) awareness of the national vaccination pro-gram’s recommendation for boys, (c) current vaccination status of sons, (d) intentions to vaccinate sons against HPV, and (e) reasons underlying vaccination decisions. The HPV awareness question was general in scope and not restricted to boys (“Do you know what HPV is and what diseases it can cause?”), while a separate item specifically assessed awareness of the national program for boys. This distinction accounts for the differing denominators reported in the results.

Attitudes and Beliefs: Parents rated the importance of factors influencing their willingness or unwillingness to vaccinate their sons using a four-point Likert scale (“strongly agree,” “agree,” “disagree,” “strongly disagree”). This section also assessed general attitudes to-ward vaccines and their importance for public health.

As a pragmatic convergent validity check, a question on measles vaccination status was included. The near-universal measles vaccination coverage observed (99.2%) is consistent with national data and supports the internal consistency of responses. However, the modified questionnaire was not formally revalidated (e.g., Cronbach’s alpha or factor analysis), which represents a methodological limitation acknowledged in the Discussion section.

### 2.4. Statistical Analysis

Descriptive statistics, including frequencies and percentages, were computed for all variables. Given the categorical nature of the data, appropriate statistical tests were ap-plied. Chi-square tests (χ^2^) were used to examine associations between parental gender and categorical variables, including variables related to knowledge, attitudes, and vaccination intentions. Fisher’s exact test was applied when expected cell counts were less than 5. Effect sizes were calculated using Cramér’s V for chi-square tests. Binary logistic regression analysis was performed to identify independent predictors of HPV awareness (aware vs. not aware), using parental gender, age group, and education level as independent variables. Logistic regression analysis for vaccination intention was not performed due to the high prevalence of positive responses and limited variability in the outcome. To further examine the association between HPV awareness and vaccination intention, 2 × 2 cross-tabulations (HPV awareness × vaccination intention) were computed separately for fathers and mothers. Statistical significance was set at *p* < 0.05 (two-tailed). All analyses were performed using the Statistical Package for the Social Sciences (SPSS) Version 25.0 (IBM Corp., Armonk, NY, USA).

### 2.5. Ethical Considerations

The study was conducted in accordance with the Declaration of Helsinki, and approved by the Institutional Review Board of medical school of University of Thessaly (or Ethics Committee) *9th /8-07-2024/ protocol number: 643*. Informed consent was obtained from all participants. Questionnaires were anonymous, and no personally identifiable information was collected. Data were stored securely and accessible only to research team members.

## 3. Results

The final analytical sample comprised 192 parents (42 fathers, 21.9%; 150 mothers, 78.1%). Table 1 presents the sociodemographic characteristics of participants stratified by gender. The majority of both fathers (95.2%) and mothers (87.3%) were of Greek origin. Regarding age distribution, fathers were more evenly distributed across age categories, with 21.4% aged 18–30 years, 40.5% aged 31–45 years, and 38.1% aged 46+ years. Mothers showed a slightly different pattern, with 36.0% aged 18–30 years, and equal proportions (32.0% each) in the two older age categories.

Educational attainment was similar between fathers and mothers, with no statistically significant differences (χ^2^ = 2.258, *p* = 0.521). Among fathers, 2.4% had completed gymnasium, 47.6% had completed lyceum, 40.5% held university degrees, and 9.5% had postgraduate qualifications. Among mothers, the distribution was 7.3%, 38.0%, 42.7%, and 12.0%, respectively. Overall, approximately 50% of both fathers and mothers had completed tertiary education or higher.

**Figure 1:**
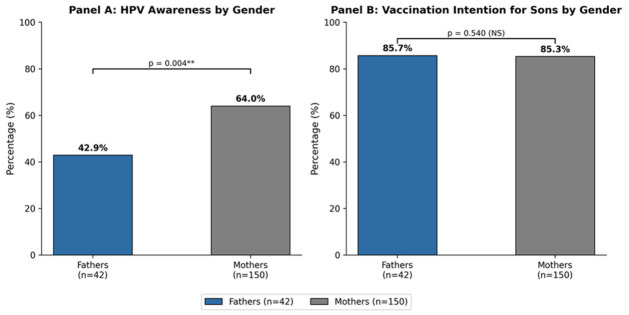
Gender differences in HPV awareness and vaccination intention for sons. Panel A: HPV awareness was significantly higher among mothers than fathers (64.0% vs. 42.9%, *p* = 0.004). Panel B: Vaccination intention for sons did not differ between groups (85.3% vs. 85.7%, *p* = 0.540). ** *p* < 0.01; NS: not significant. Blue bars = fathers (*n* = 42); grey bars = mothers (*n* = 150).

Table 2 presents comparisons of HPV knowledge and awareness between fathers and mothers. A striking gender difference emerged in basic HPV awareness. When asked whether they knew about HPV and its related diseases, only 42.9% (18/42) of fathers responded affirmatively, compared with 64.0% (96/150) of mothers. Half of fathers (50.0%, 21/42) explicitly stated they did not know about HPV, compared with 35.3% (53/150) of mothers, Figure 1. This difference was statistically significant (χ^2^ = 10.907, *p* = 0.004), indicating that mothers have substantially higher baseline HPV awareness.

Knowledge about the national vaccination program was uniformly low among both parents, though slightly higher among mothers. Only 9.5% (2/21) of fathers were aware that the national immunization program recommends and offers free HPV vaccination for boys aged 9–12 years, compared to 15.5% (16/103) of mothers. The majority of both fathers (66.7%, 14/21) and mothers (68.9%, 71/103) were unaware of this recommendation. These findings highlight a critical gap in public health communication, as the national vaccination program was relatively new at the time of data collection.

### 3.1. Vaccination Intentions and Attitudes

Despite the significant gender gap in HPV knowledge, vaccination intentions were similar between fathers and mothers (Table 3). When asked whether they planned to vaccinate their sons against HPV, 85.7% (36/42) of fathers and 85.3% (128/150) of mothers responded affirmatively. Only 9.5% of fathers and 12.7% of mothers reported they did not intend to vaccinate, while small proportions reported their sons were already vaccinated (4.8% and 2.0%, respectively). These differences were not statistically significant (χ^2^ = 1.232, *p* = 0.540), indicating that vaccination intention does not differ by parental gender. This finding is particularly noteworthy: fathers with lower HPV knowledge were as willing to vaccinate their sons as more knowledgeable mothers. This suggests that knowledge gaps do not necessarily translate into vaccine hesitancy, and that fathers’ lower awareness may reflect barriers to information access rather than fundamental opposition to vaccination. General vaccine attitudes were also largely similar between fathers and mothers. When asked whether vaccines are important for public health, 63.4% of fathers and 70.9% of mothers agreed or strongly agreed, with no significant gender difference. Regarding the statement that “vaccines have eliminated many diseases,” 100% of fathers agreed or strongly agreed, compared with 95.4% of mothers. These findings indicate strong pro-vaccine attitudes among both parents, providing a favorable foundation for promoting HPV vaccination.

Regarding vaccination coverage, the following findings were recorded. A total of 99.2% of participants had vaccinated their sons against measles with at least one dose, while 80% had completed the recommended two-dose measles vaccination schedule. Among respondents, 59% (*n* = 113) had already vaccinated their sons against the human papillomavirus (HPV) with one or two doses. Of these, (*n* = 24), 19.2% had received only a single dose, whereas 71.2% (*n* = 89) had completed the two-dose vaccination regimen.

Among parents who intended to vaccinate their sons, the most frequently cited reasons were similar across genders. Both fathers emphasized the importance of equal gender responsibility in preventing sexually transmitted infections (fathers: 88.9%; mothers: 92.2%) and in protecting against cancer (fathers: 86.1%; mothers: 88.3%). The perception that vaccination is important because “it’s the right thing to do” was also commonly endorsed (fathers: 83.3%, mothers: 85.9%).

Among parents who were uncertain or unwilling to vaccinate, the most commonly cited barriers were also similar between genders. Fear of adverse reactions was the primary concern for both fathers (30.0%) and mothers (32.1%). Insufficient knowledge about HPV and the vaccine was the second most common reason (fathers: 25.0%, mothers: 26.0%). These findings suggest that while fathers have lower baseline knowledge, both parents identify knowledge gaps as a key barrier to vaccination acceptance.

### 3.2. Association Between HPV Knowledge and Vaccination Intention

The association between HPV awareness and vaccination intention was examined by cross-tabulation, separately for fathers and mothers. No statistically robust association between HPV awareness and vaccination intention could be demonstrated in either group. Among fathers, Fisher’s exact test yielded *p* = 0.122 (NS), with statistical power further limited by the small subgroup size (*n* = 42). Among mothers, interpretation of the chi-square test was constrained by the very high overall vaccination intention rate (85.3%), which produced limited variability in the outcome and may inflate test statistics; formal inference on this association should therefore be treated with caution. Overall, while descriptive patterns suggest potential differences between fathers and mothers in the knowledge–intention relationship, formal statistical testing did not demonstrate a robust association between HPV awareness and vaccination intention in either group, most likely reflecting the high prevailing intention rates and limited outcome variability in this sample.

Binary logistic regression was performed to identify independent sociodemographic predictors of HPV awareness. Parental gender (female sex) was the only significant independent predictor (OR = 2.26, 95% CI: 1.12–4.58, *p* = 0.024), after controlling for education level and age group; neither education (OR = 1.40, *p* = 0.267) nor age group (OR = 0.75, *p* = 0.127) reached significance (Table 4). A logistic regression model with vaccination intention as the dependent variable was not pursued as a primary analysis, given the limited variability in the outcome (85–90% intention rate) and the small father subgroup, which would render any inferential estimates unreliable. These regression results confirm that gender differences are confined to HPV knowledge acquisition, while the decision to vaccinate cannot be reliably modeled with the available sample size and outcome distribution.

## 4. Discussion

This study provides novel evidence of significant gender differences in HPV knowledge among Greek parents, while revealing equal vaccination intentions across genders. Three key findings emerge: (1) fathers have substantially lower awareness and knowledge about HPV compared to mothers, with a 21-percentage-point gap in basic HPV awareness, (2) despite this knowledge gap, fathers demonstrate equal willingness to vaccinate their sons against HPV, and (3) both fathers and mothers have low awareness of the national vaccination program for boys and identify insufficient knowledge as a barrier to vaccination.

### 4.1. Fathers’ Knowledge Gap: A Reflection of Gendered Health Information Access

The finding that only 42.9% of fathers knew about HPV, compared to 64.0% of mothers (*p* = 0.004), aligns with broader patterns in health literacy research showing that mothers typically serve as family health information brokers [30,31]. This gender gap likely reflects structural and cultural factors rather than differential interest in children’s health. Mothers more frequently access pediatric healthcare settings, where HPV vaccination information is typically provided [32]. In Greece, as in many countries, mothers predominate at well-child visits and vaccination appointments, creating systematic differences in information exposure [33].

Our findings are consistent with international research showing lower HPV awareness among fathers. Studies from the United Kingdom, United States, and Canada have similarly reported that fathers have less knowledge about HPV and the vaccine compared to mothers [23,34,35]. However, our study extends these findings by quantifying the magnitude of the gender gap in a Southern European context where gender-neutral vaccination was newly implemented.

Importantly, fathers’ lower knowledge appears to reflect barriers to information access rather than active disinterest. When asked about their concerns, fathers cited insufficient knowledge at rates similar to those of mothers (25.0% vs. 26.0%), suggesting awareness of their knowledge gaps. Moreover, fathers generally demonstrated strong pro-vaccine attitudes, with 100% agreeing that vaccines have eliminated diseases. These findings suggest that fathers are receptive to vaccination but have been systematically excluded from health information channels.

### 4.2. Equal Vaccination Intentions Despite Knowledge Gaps

Perhaps the most striking finding is that fathers’ lower knowledge did not translate to lower vaccination intentions. Both fathers (85.7%) and mothers (85.3%) expressed high willingness to vaccinate their sons, rates that exceed those reported in some previous Greek studies [36]. This dissociation between knowledge and intention challenges the traditional knowledge–attitude–behavior model often applied to vaccination decisions [37]. (Despite a 21-point knowledge gap, both groups reached identical vaccination intentions, suggesting knowledge gaps reflect information access barriers rather than vaccine hesitancy.)

Several explanations may account for this finding. First, fathers may defer to mothers’ expertise on health matters while maintaining trust in vaccination generally. Second, high vaccination intentions may reflect strong endorsement of preventive healthcare in principle, even when specific knowledge is limited. Third, the recent policy change making HPV vaccination free for boys may have increased perceived legitimacy and social desirability of vaccination.

Our findings contrast with some previous research suggesting that parental knowledge predicts vaccination intentions [38,39]. However, other studies have similarly found that high vaccination intentions can coexist with knowledge gaps, particularly when general pro-vaccine attitudes are strong [40,41]. The Greek context may be particularly relevant here: Greece has historically maintained high childhood vaccination coverage, and vaccine confidence remained relatively strong even during the economic crisis [42].

### 4.3. Implications for Gender-Inclusive Health Promotion

These findings have important implications for HPV vaccination promotion strategies. Current public health communications appear to perpetuate gender disparities by targeting mothers almost exclusively. Pediatric healthcare materials, vaccine information campaigns, and school-based programs typically address “parents” while depicting mothers or using feminine pronouns [43]. Healthcare providers similarly direct vaccination discussions primarily to mothers [18].

To capitalize on fathers’ positive vaccination intentions while addressing their knowledge needs, several strategies merit consideration:

1. Father-Targeted Information Campaigns: Public health agencies should develop communications specifically designed to reach fathers through channels they access, such as workplaces, sports settings, and social media platforms popular with men. Messages should acknowledge fathers’ active parenting roles and their importance in vaccination decisions.
2. Inclusive Healthcare Practices: Pediatric and primary care providers should actively engage both parents in vaccination discussions, including offering evening or weekend appointment times that accommodate working fathers. Vaccination reminder systems should include multiple parent contacts.
3. Male-Friendly Information Format: HPV education materials should include content relevant to fathers, such as information about HPV-related cancers affecting men, the protective benefits for future sexual partners, and the role of fathers as health decision-makers. Materials should avoid assumptions that mothers are sole decision-makers.
4. School-Based Programs: Given that both parents showed high vaccination intentions, school-based vaccination programs with opt-in rather than opt-out consent may be particularly effective, requiring active engagement from both parents.
5. Leveraging Male Healthcare Encounters: Since fathers may have limited pediatric healthcare contact, HPV vaccination information could be provided during fathers’ own healthcare visits, including occupational health services.

### 4.4. Addressing Low Awareness of National Programs

The finding that only 9.5% of fathers and 15.5% of mothers knew about the national vaccination program recommendation for boys represents a critical public health communication failure. This low awareness likely reflects several factors: (1) the recommendation for boys was relatively recent at the time of data collection; (2) public health messaging may have continued to emphasize cervical cancer prevention in girls; (3) the economic crisis and COVID-19 pandemic disrupted routine health communications; and (4) parents of boys may not have perceived HPV vaccination as relevant to their children.

Improving awareness of gender-neutral vaccination policies requires sustained, multi-channel communication efforts. Greece’s experience mirrors challenges faced in other countries implementing gender-neutral vaccination, where awareness campaigns lagged behind policy changes [44,45]. Successful awareness campaigns in Australia and the United Kingdom have demonstrated the importance of clear, consistent messaging about benefits for both genders and addressing misperceptions that HPV primarily affects women [46,47].

This study has several strengths, including a direct comparison of fathers and mothers using standardized measures, an adequate sample size to detect clinically meaningful differences, and a focus on a timely public health issue in Greece. The study provides important baseline data on parental attitudes during Greece’s transition to gender-neutral vaccination.

However, limitations must be acknowledged. First, the study employed convenience sampling in primary healthcare waiting rooms, which may limit generalizability and have contributed to the overrepresentation of mothers. Second, the relatively small number of fathers (*n* = 42) reduced the precision of subgroup analyses. Third, the high prevalence of positive vaccination intention limited the variability of the outcome, constraining inferential analyses. Fourth, self-reported data may be subject to social desirability bias, potentially inflating vaccination intentions. Fifth, the modified questionnaire was not formally revalidated in this form [26,27]. Additionally, the possibility that both parents from the same family participated cannot be excluded, which may have influenced the independence of responses.

Future research should employ probability sampling, include larger and more balanced father samples, and incorporate validated instruments, as well as designs that allow for within-family comparisons. Further investigation into the pathways through which fathers access health information would help clarify the mechanisms underlying the observed knowledge gap. Future studies should also include parallel assessment of vaccination intentions for daughters, which would allow direct within-family comparison of parental attitudes by child sex.

## 5. Conclusions

This study demonstrates that Greek fathers have significantly lower HPV knowledge than mothers but equal willingness to vaccinate their sons. Rather than reflecting vaccine hesitancy, fathers’ knowledge gaps may reflect differences in exposure to health information, particularly through healthcare settings more frequently attended by mothers. Although this study did not directly assess pathways of information access, this interpretation is consistent with existing literature and should be explored further in future re-search.

Parents (mothers and fathers) play a pivotal role in the implementation of vaccination programs and in ensuring children’s adherence to vaccination recommendations, particularly in age groups where children are not able to make vaccination decisions independently. The findings of the present study the positive attitudes of fathers about HPV vaccination of sons further support the establishment of educational and informational programs that could be implemented to promote a unified approach and provide parental guidance and counseling regarding HPV vaccinations.

To optimize HPV vaccination coverage in Greece, public health programs should actively engage fathers through targeted communication strategies and inclusive healthcare practices that recognize their role in family health decisions. Addressing fathers’ information needs, while leveraging their positive attitudes toward vaccination, may contribute to improved health literacy and more equitable vaccination uptake.

As gender-neutral HPV vaccination strategies continue to expand, understanding parental decision-making dynamics becomes increasingly important. Engaging both parents through tailored and inclusive approaches represents a practical step toward improving vaccination uptake.

## Figures and Tables

**Table 1:** Sociodemographic Characteristics Stratified by Parental Gender (N = 192).

Characteristic	Fathers (*n* = 42)	Mothers (*n* = 150)	*p*-Value
Age Group			0.203
18–30 years	9 (21.4%)	54 (36.0%)	
31–45 years	17 (40.5%)	48 (32.0%)	
46+ years	16 (38.1%)	48 (32.0%)	
Education Level			0.521
Secondary (Gymnasium)	1 (2.4%)	11 (7.3%)	
Secondary (Lyceum)	20 (47.6%)	57 (38.0%)	
University	17 (40.5%)	64 (42.7%)	
Postgraduate	4 (9.5%)	18 (12.0%)	
Country of Origin			0.178
Greece	40 (95.2%)	131 (87.3%)	

**Table 2:** Gender Differences in HPV Knowledge and Awareness.

Knowledge Item	Fathers’ n/N	Fathers %	Mothers n/N	Mothers %	*p*-Value
Aware of HPV existence	18/42	42.9	96/150	64.0	0.004 **
Aware of the national program	2/18	11.1	16/96	16.7	NS
HPV is sexually transmitted	15/18	83.3	82/96	85.4	NS

Note: Row 2–3 show responses among those aware of HPV (*n* = 18 fathers, *n* = 96 mothers) ** *p* < 0.01; NS: not significant.

**Table 3:** Vaccination Intentions and Attitudes by Parental Gender.

Item	Fathers’ n/N	Fathers %	Mothers n/N	Mothers %	*p*-Value
Intend to vaccinate son	36/42	85.7	128/150	85.3	0.540
Vaccines are important for public health	26/41	63.4	105/148	70.9	0.463
Vaccines eliminated diseases	42/42	100.0	143/150	95.3	0.350
Fear of adverse reactions (barrier)	3/10	30.0	10/31	32.3	1.000

Note: The Last row shows respondents who did not intend to vaccinate or were uncertain.

**Table 4:** Binary Logistic Regression: Independent Predictors of HPV Awareness (N = 192).

Variable	OR	95% CI	*p*-Value
Female sex (ref: male)	2.26	1.12–4.58	0.024 *
Education level	1.40	0.77–2.54	0.267
Age group	0.75	0.52–1.08	0.127

* *p* < 0.05. OR: Odds Ratio; CI: Confidence Interval. Reference category for female sex: male. The 95% CI for education level and age group are estimated from the reported OR and *p*-values.

## Data Availability

The raw data supporting the conclusions of this article will be made available by the authors on request.
